# High Performance of Metallic Thin Films for Resistance Temperature Devices with Antimicrobial Properties

**DOI:** 10.3390/s22197665

**Published:** 2022-10-09

**Authors:** Arthur L. R. Souza, Marcio A. Correa, Felipe Bohn, Helder Castro, Margarida M. Fernandes, Filipe Vaz, Armando Ferreira

**Affiliations:** 1 Centro de Física das Universidades do Minho e do Porto (CF-UM-UP), Universidade do Minho, 4710-057 Braga, Portugal; 2 Departamento de Física, Universidade Federal do Rio Grande do Norte, Natal 59078-900, RN, Brazil; 3 LaPMET—Laboratório de Física para Materiais e Tecnologias Emergentes, Universidade do Minho, 4710-057 Braga, Portugal

**Keywords:** temperature sensors, antimicrobial, titanium-copper, thin films

## Abstract

Titanium-copper alloy films with stoichiometry given by Ti${}_{1-x}$Cu${}_{x}$ were produced by magnetron co-sputtering technique and analyzed in order to explore the suitability of the films to be applied as resistive temperature sensors with antimicrobial properties. For that, the copper (Cu) amount in the films was varied by applying different DC currents to the source during the deposition in order to change the Cu concentration. As a result, the samples showed excellent thermoresistivity linearity and stability for temperatures in the range between room temperature to 110 °C. The sample concentration of Ti${}_{0.70}$Cu${}_{0.30}$ has better characteristics to act as RTD, especially the $\alpha_{TCR}$ of 1990 $\times \;10^{-6}$ ${}^{{}^{\circ}}$C${}^{-1}$. The antimicrobial properties of the Ti${}_{1-x}$Cu${}_{x}$ films were analyzed by exposing the films to the bacterias *S. aureus* and *E. coli*, and comparing them with bare Ti and Cu films that underwent the same protocol. The Ti${}_{1-x}$Cu${}_{x}$ thin films showed bactericidal effects, by log${}_{10}$ reduction for both bacteria, irrespective of the Cu concentrations. As a test of concept, the selected sample was subjected to 160 h reacting to variations in ambient temperature, presenting results similar to a commercial temperature sensor. Therefore, these Ti${}_{1-x}$Cu${}_{x}$ thin films become excellent antimicrobial candidates to act as temperature sensors in advanced coating systems.

## 1. Introduction

Temperature sensors are devices having a broad range of technological applicability in different branches of industries, such as electronics, automotive, and aerospace [1,2]. The most common types of temperature sensors include thermistors, Resistance Temperature Detectors (RTDs), thermocouples, and semiconductor-based sensors [3,4,5]. They can be categorized into contact and non-contact types by temperature measuring method. The non-contact type sensors, which are based on thermal emission of electromagnetic radiation, are used mainly in research and development fields. The contact type sensor includes thermocouples, thermistors, and RTDs which are based on the Seebeck effect, temperature-sensitive electrical resistance, and positive/negative temperature coefficient of electrical resistance, respectively, [6,7,8,9,10,11]. At present, thermocouples and thermistors are most widely used in industry. A thermistor has high sensitivity, fast time response, and low price, but it suffers from limited operating temperature range and nonlinear resistance versus temperature response. Thin film sensors have received great interest because of their lower consumption of precious materials and high productivity owing to the existing high technology used in the semiconductor industry. In particular, RTD is a thermoresistor whose electrical resistance varies linearly with temperature [2,4,5]. The sensor has high linearity, stability, and a wide operating temperature range, but has a high price and slow response time. In the past, most RTDs were wire type, which used film platinum wire covered in an insulated tube as the sensing element, but thin film types of RTDs are now replacing the wire type because of their small dimensions and short response time.

Additionally, RTD’s sensors based on thin film technology bring advantages compared to bulk ones, for example, they have high sensitivity, stability, and small dimensions that can be produced with high reproducibility and low-cost [12]. Moreover, sensors based on thin film make easy integration with complex systems in which the temperature dependence is fundamental for effects, such as spintronics [13], piezo- and thermo-resistivity properties [14], and antibacterial coatings [15].

In this context, it is increasingly important the development multifunctional sensors based on thin films for the new generation of smart devices. Traditionally, Platinum (Pt) is the most employed material to compose RTD devices [4,16], despite its high cost. For measurements until ≈300 °C, metals such as Copper (Cu) or Nickel (Ni) are well-established, and they are exciting alternatives to replace Pt, presenting strong linearity and stability in a wide temperature range.

The application of smart coatings capable of preventing either viral or bacterial infections is a timeless high-demand research topic, in particular in pandemic scenarios. For this, a widely used strategy is using metal-ion-based thin films with antimicrobial properties to develop antibacterial coatings [17]. For instance, Copper (Cu), and the ion Cu${}^{2+}$, are excellent candidates to act as an antimicrobial agent [18,19] in a metal-ion-based thin film due to the cytocompatibility and lower toxicity [20].

Therefore, the engineering of nanostructured thin films based on Ti and Cu materials brings the possibility to reach sensors with multifunctional features, where resistance to corrosion, thermoresistivity response, and biocompatibility are present [21,22]. The combination of a thermal functionalized with antimicrobial characteristics can open interesting solutions for high-traffic surfaces [23,24,25,26]. Remarkably, the integration between temperature sensors and antimicrobial response is absent in the literature.

This work explores the role of the Cu addition into a Ti matrix on the thermosensitive and the antibacterial response of Ti${}_{1-x}$Cu${}_{x}$ films produced by DC co-sputtered thin films. For this purpose, a set of Ti${}_{1-x}$Cu${}_{x}$ thin films were prepared and then characterized in terms of their morphological, structural, thermosensitive, and antimicrobial properties. Irrespective of the Ti${}_{1-x}$Cu${}_{x}$ concentration, we observed great stability in the thermoresistive response of the films, which turns this system into an interesting alternative to functionalizing the surfaces. However, in this study, we go beyond the characterization of thin films and present a prototype to reach the thermosensitive signal by using an operational amplifier to measure the temperature along the time.

## 2. Materials and Methods

Magnetron sputtering were used to deposit Ti${}_{1-x}$Cu${}_{x}$ films form Ti and Cu targets with 99.99% purity. The base pressure was 2.5 × 10${}^{-6}$ mbar, during the deposition, 4.5 × 10${}^{-3}$ mbar of Argon (Ar) was inserted in the chamber. The films were growth co-sputtering using a DC source, in which a fixed current of 200 mA for the Ti target was considered. On the other hand, for the Cu target the DC currents were 5, 10, and 15 mA ensuring the change in film stoichiometry. All the depositions were done during 20 min under amorphous glass and Si (100) substrates. After each deposition, the samples were annealed, in vacuum, at 250 °C for 60 min and with a pressure of 2.5 × 10${}^{-6}$ mbar to improve the structural properties of the samples.

X-ray diffraction (XRD) and Scanning Electron Microscopy (SEM-FEG) (Center for Electron Nanoscopy, Lyngby, Denmark) measurements were used to verify the structure and morphology of our samples. In particular, the XRD was measured using a Bruker D8 Discover diffractometer (Bruker, Billerica, Massachusetts, EUA), with Cu-k${}_{\alpha }$ radiation in the θ−2θ configuration. The SEM-FEG was used to verify the top and cross-section views of the samples, here we realized the measurements using a NanoSEM—FEI Nova 200 (FEG/SEM). The film composition was measured from Electron Dispersive X-ray Spectroscopy (EDS) technique with an EDAX—Pegasus X4M (EDS/EBSD).

For antimicrobial characterization, Gram negative *Escherichia coli* ATCC${}^{®}$ 8739^™^ and *Staphylococcus aureus* ATCC${}^{®}$ 6538^™^ were purchased from the American Type Culture Collection (LGC Standards S.L.U, Spain). The bacterial pre-inoculum was prepared from a single colony of the corresponding stock bacterial culture, which was resuspended in nutrient broth (NB) and then incubated overnight at 37 °C, and 110 rpm. After 20 h, the bacteria were harvested by centrifugation at 4500 rpm for 5 min and resuspended in NaCl 0.9% (*w*/*v*) twice. The *E. coli*-cultures optical density (OD) was adjusted to OD = 0.26 and the *S. aureus*-cultures OD was adjusted to 0.2 with NaCl 0.9% (*w*/*v*) measured at 600 nm, giving rise to a working inoculum of approximately $1\times 10^{8}$ colony forming units (CFU) per mL.

The bactericidal activity was assessed according to the standard shake flask method (ASTM-E2149-01) with some modifications. This method provides quantitative data for measuring the reduction rate in the number of bacteria colonies formed, converted to the average colony-forming units per milliliter of buffer solution in the flask (CFU/mL). To evaluate the potential of the materials to eradicate *E. coli* and *S. aureus*, samples with 2×2 cm${}^{2}$ in size, previously sterilized under UV light for 30 min each side, were placed in contact with each bacterial inoculum (1 mL of working inoculum) in a 15 mL falcon tubes. The tubes were then placed under vigorous agitation (220 rpm) at 37 °C for 2 h. The bacterial solution in contact with the material and respective controls was then removed, and the surviving colonies were quantified by serially diluting (1:10) in sterile buffer solution, plated on a plate count NB agar, and further incubated at 37 °C for 24 h. Antimicrobial activity is reported in terms of bacteria log reduction calculated as the ratio between the number of surviving bacteria after and before contact with the materials according to the following equation,

(1) $$BR\left(\%\right)=log_{10}\left(A\right)-log_{10}\left(B\right)$$

where *BR* is the bacterial $log_{10}$ reduction, *A* and *B* are the average number of bacteria before and after contact with the samples, respectively. The results were further expressed as $log_{10}$ reduction by calculating the *BR* (%). All antibacterial data represent mean values 3 independent assays ± SD (n=3).

The Live/Dead BacLightTM Bacterial Viability Kit (Invitrogen, USA) was used to qualitatively evaluate the viable and non-viable bacteria adhered to the material. The samples that were previously in contact with bacterial inoculum for the CFU assay were then washed with PBS and stained for 15 min with a mixture of 1.5 μL green-fluorescent SYTO 9 and red-fluorescent propidium iodide in the dark. Finally, the imaging of the samples was performed using a fluorescence microscope (Olympus BX63F2 microscope). The representative images were taken at a magnification of 100×.

The thermoresistive response was measured using a Keithley—2700 series high-precision multimeter and a Linkam LTS420 system to control the temperature applied to the films. The calculation of the Temperature Coefficient of Resistance ($\alpha_{TCR}$) was obtained through

(2) $$R_{f}=R_{i}\left(1+\alpha_{TCR}\left(T_{f}-T_{i}\right)\right)$$

where *R*${}_{f}$ and *R*${}_{i}$ is the final and initial film’s resistance and *T*${}_{f}$ and *T*${}_{i}$ is the final and initial temperature.

**Figure 1:**
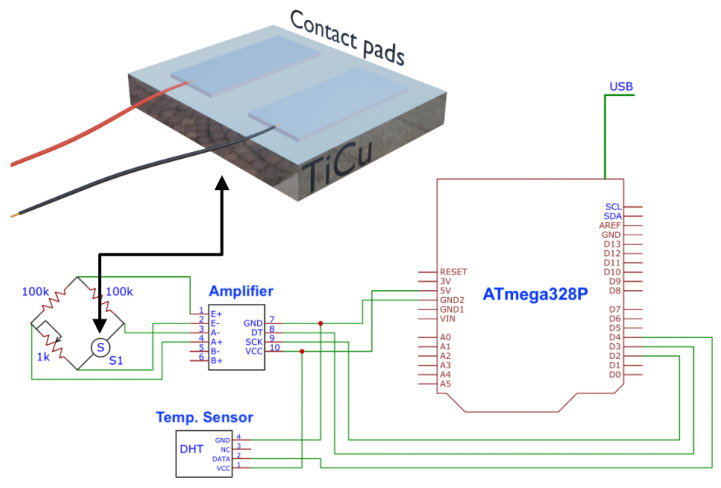
Block diagram of the thermoresistive sensor readout circuit. At the top, the sample structure with the Titanium pads is depicted.

To read the signal of the Ti${}_{1-x}$Cu${}_{x}$ thin film developed and a comparative standard sensor (DHT11 temperature sensor), an electronic reading system (Figure 1) was made based on an Arduino protocol and connected both to the microcontroller that allows us to be able to read the changes in the resistance of the resistive sensor developed. The system was plugged into a universal serial bus (USB) port. To calibrate the circuit with the sensor, a potentiometer (100 KΩ) was used in the Wheatstone Bridge.

## 3. Results and Discussion

**Figure 2:**
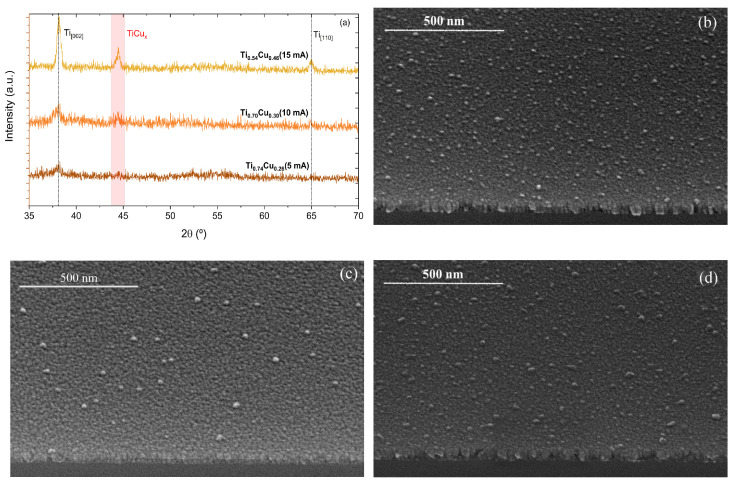
(**a**) X-ray diffraction of produced thin films annealed at 250 ${}^{{}^{\circ}}$C. The peaks are indexed through ICSD-43416 for Ti and ICSD-103128, ICSD-629379I, and CSD-107712 for Ti${}_{1-x}$Cu${}_{x}$. Representative tilted top-view SEM. micrographs for the samples prepared with (**b**) 5 mA, (**c**) 10, and (**d**) 15 mA.

Figure 2a shows the XRD results for the studied thin films. The thin films have a hexagonal Ti structure, characterized by the (002) and (110) peaks (ICSD-43416) located at 2θ≈38.4${}^{{}^{\circ}}$ and 2θ≈65.0${}^{{}^{\circ}}$, respectively. With the increase of DC current set to the Cu target, it is possible to observe the peaks, and highlight region in 2θ≈44.4${}^{{}^{\circ}}$, which can be associated with TiCu${}_{x}$ phases, such as TiCu (111) (ICSD-103128), TiCu${}_{2}$ (311) (ICSD-629379), or TiCu${}_{3}$ (121) (ICSD-107712), due to their similar angular positions.

Figure 2b–d shows the representative SEM. micrographs, in a tilted top-view, for the samples prepared using distinct currents set in the power source. From Figure 2b–d, it is possible to observe a considerable number of Cu islands on the surface of the samples, which changes the roughness and the homogeneity in the Cu distribution in the Ti matrix, and consequently influences the electrical response due to the scattering processes [27]. From the EDS measurements performed during SEM characterization, it is possible to observe an increase in the Cu amount (at.%) in the samples. Table 1 summarize the EDS results.

**Figure 3:**
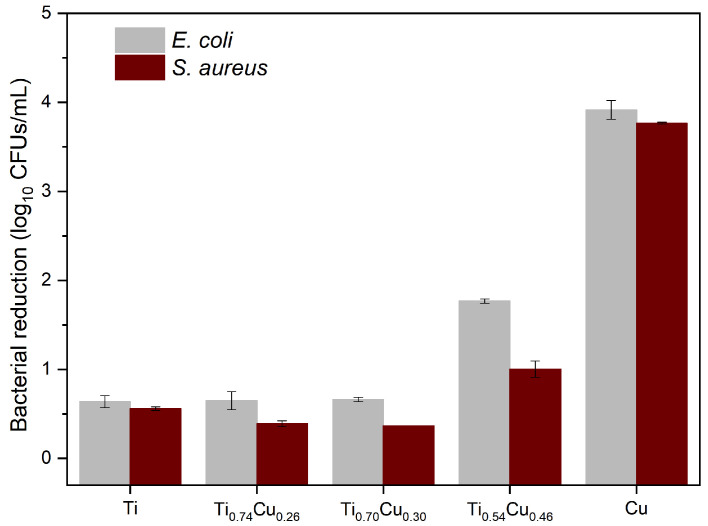
Antimicrobial activity of the materials against *E. coli* and *S. aureus*, measured in $log_{10}$ reduction of CFUs. The results represent three individual measurements.

As observed in Figure 3, the Ti-based materials/coatings containing increasing concentrations of Cu were tested against two bacteria, one Gram positive *S. aureus* and one Gram negative *E. coli*. Saline solution containing bacteria was placed in contact with the material for 2 h, and the live bacteria in the medium was quantified by CFUs. The control samples, i.e., coatings comprised of Ti or Cu alone, have shown that the antimicrobial activity is due to the presence of Cu. Ti sample possessed a minor effect on both bacteria while the cupper coating induced the highest bacterial $log_{10}$ reduction of approximately 4 on both bacteria. Among the hybrid samples, only the Ti${}_{0.54}$Cu${}_{0.46}$ induced significant bactericidal effect (approximately 2 $log_{10}$ reductions for *E. coli* and 1 $log_{10}$ reduction for *S. aureus*), while the other, Ti${}_{0.74}$Cu${}_{0.26}$ and Ti${}_{0.70}$Cu${}_{0.30}$, showed bacterial reduction values comparable to the Ti sample. The copper action mechanism against bacteria depends on the release of copper ions into the medium, which inhibits cell respiration, induces bacterial membrane disruption, or destroys the intracellular DNA and RNA [28]. This property is important for avoiding the occurrence of resistance since it destroys the machinery for mutation [28].

**Figure 4:**
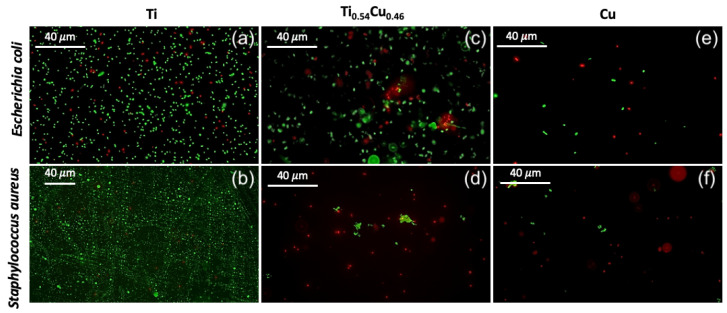
Fluorescence microscopy images of *E. coli* and *S. aureus* after 2 h in contact with the material. Live cells are represented in green and dead cells in red. (**a**,**b**) Results obtained for Ti film. (**c**,**d**) Results obtained for representative Ti${}_{0.54}$Cu${}_{0.46}$ film. (**e**,**f**) Results for Cu film.

The material subjected to the presence of bacterial inoculum for 2 h was then analyzed by fluorescence microscopy using a live/dead kit, as depicted in Figure 4a–f. For clarity, in this analysis, we considered only three compounds, pure Ti (Figure 4a,b), Ti${}_{0.54}$Cu${}_{0.46}$ sample (Figure 4b,c), and pure Cu (Figure 4d,e), once presented the best antimicrobial results, as discussed before. As expected, more bacteria were found at the surface of the material comprising only Ti, while the coating comprised of Ti${}_{0.54}$Cu${}_{0.46}$ possessed fewer cells and induced important cell death (cells stained in red), corroborating the results from CFU analysis. The control made only of bare Cu presented an even lower quantity of cells at the surface, most of them being dead. The results were transversal to both Gram positive and Gram negative bacteria.

The antimicrobial properties of the coatings were thus related to the presence of copper in the coating, as Ti control possessed only residual $log_{10}$ bacterial reduction and presented only live cells at the surface of the material. This was expected since Ti is usually a biologically inert compound [29]. Nevertheless, the higher the amount of copper, the better the bactericidal activity, measured either in contact with bacteria-rich aqueous-based medium (Figure 3) or on the surface of the samples (Figure 4). Copper has been known for health applications since ancient times when copper oxides were commonly used to treat skin infections [30]. In fact, copper compounds have been reported to possess numerous biological activities such as anti-proliferative, anti-inflammatory, and antimicrobial, among others [31]. The most interesting property is the bactericidal activity since the mechanism of action of copper, as mentioned above, includes damaging the bacterial DNA and RNA which prevents the bacteria from acquiring resistance to copper. This feature is very appealing due to the ever-growing antimicrobial resistance, considered one of the serious concerns in health for the future. The research on copper for antimicrobial applications has been growing since it has been recognized by the United States Environmental Protection Agency (US EPA) as the first antimicrobial metal in 2008. Copper-containing materials have been reported to possess potent antimicrobial activity [32] being able to kill 99.9% of bacteria [33] in 2 h contact and even induce a 7 to 8 $log_{10}$ reduction in only 1 h [34].

**Figure 5:**
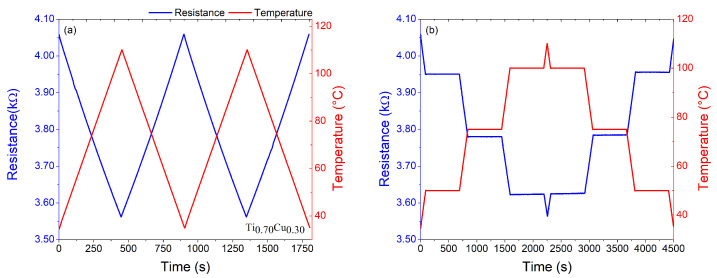
(**a**) Electrical resistance measured under temperature cycles for the Ti${}_{0.70}$Cu${}_{0.30}$ sample. (**b**) Electrical resistance stability for 50, 75, and 100 ${}^{{}^{\circ}}$C for periods of 10 min for the Ti${}_{0.70}$Cu${}_{0.30}$ sample.

**Figure 6:**
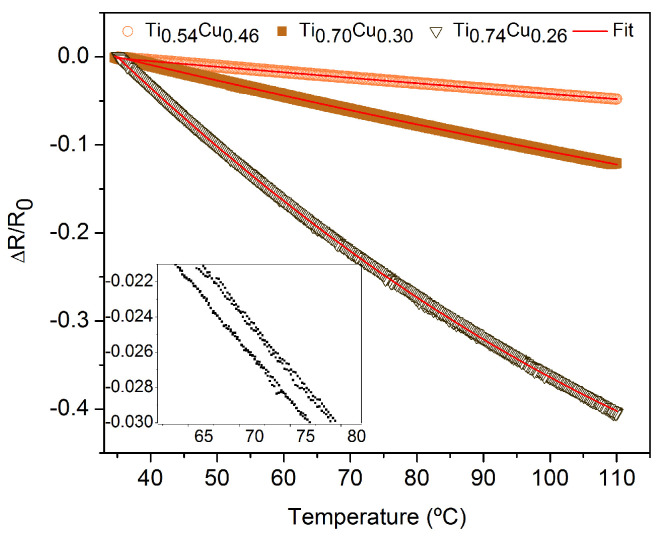
$\frac{\Delta R}{R_{0}}$ as a function of the temperature. The inset depicts the curve for Ti${}_{0.70}$Cu${}_{0.30}$ sample before the annealing.

Taking into account the main purpose of this work, i.e., to explore the role of the Cu addition into the Ti matrix on the structural and thermosensitive response, it is imperative to analyze the electrical response of the films as a function of temperature. For that, a well-established protocol was employed for the prepared samples. First, the samples were subjected to two heating and cooling cycles with a rate of 10 °C/min, from room temperature to 110 °C. Second, in order to analyze the electrical signal stability, the samples were heated and cooled at the same rate, but with temperature steps holder of 10 min at each temperature of 50, 75, and 100 °C, as shown in Figure 5a,b. It is important to point out that the maximum temperature studied here is related to the real applicability of our sensor element in the environment or the human body, which rarely reaches temperatures above 50 °C. The sensitivity of the samples ($\alpha_{TCR}$) was calculated using Equation (1) and by the best fit of $\frac{\Delta R}{R_{0}}$ as a function of temperature, as depicted in Figure 6. The inset in Figure 6 shows a similar plot for the Ti${}_{0.70}$Cu${}_{0.30}$ (10 mA) sample before the annealing, where it is possible to observe very low $\frac{\Delta R}{R_{0}}$ values. This feature brings to light the importance of the annealing procedure to improve the thermosensitive response of the system.

As we can see in Figure 5a,b, in the range of temperature between 35 ${}^{{}^{\circ}}$C and 110 °C, the electrical resistance for the Ti${}_{0.70}$Cu${}_{0.30}$ sample has an linear behavior with the temperature variation and high $\alpha_{TCR}$ of (1990 ± 10) $\times \;10^{-6}$ ${}^{{}^{\circ}}$C${}^{-1}$, when compared with the traditional bulk platinum—3500 $\times \;10^{-6}$ ${}^{{}^{\circ}}$C${}^{-1}$ [35], Copper—4270 $\times \;10^{-6}$ ${}^{{}^{\circ}}$C${}^{-1}$, Nickel—6720 $\times \;10^{-6}$ ${}^{{}^{\circ}}$C${}^{-1}$, and Carbon nanotube films—1030 $\times \;10^{-6}$ ${}^{{}^{\circ}}$C${}^{-1}$ [36], the Ti${}_{0.70}$Cu${}_{0.30}$ thin film proves to be an interesting alternative as a thermal sensor component. Decreasing the amount of copper in the Ti${}_{0.74}$Cu${}_{0.26}$, the $\alpha_{TCR}$ increased to (8770 ± 10) $\times \;10^{-6}$ ${}^{{}^{\circ}}$C${}^{-1}$. However, the electrical resistance shows exponential behavior with the temperature (see the Supplementary Material). On the other hand, increasing the amount of copper in the Ti${}_{0.54}$Cu${}_{0.46}$ keeps the linear behavior of the electrical resistance with temperature (see Supplementary Material), although the $\alpha_{TCR}$ drops to (676 ± 7) $\times \;10^{-6}$ ${}^{{}^{\circ}}$C${}^{-1}$. Figure 5b shows that when the Ti${}_{0.70}$Cu${}_{0.30}$ film was subjected to a temperature step holder, the results show good stability and no visible oxidation. The $\frac{\Delta R}{R}$ slope variation as a function of the temperature depicted in Figure 6 exhibits similar behavior to the negative thermistors and does not show hysteresis during the consecutive heating and cooling cycles, indicating the possibility of these materials doped with copper to be used as RTDs for temperature ranges from room temperature to 100 ${}^{{}^{\circ}}$C. The inset of this figure shows the amplified $\frac{\Delta R}{R}$ as a function of temperature for the Ti${}_{0.70}$Cu${}_{0.30}$ film before the annealing. From the inset is possible to observe a hysteretic behavior of the thermosensitive response of the film, features that were eliminated before the annealing procedure.

**Figure 7:**
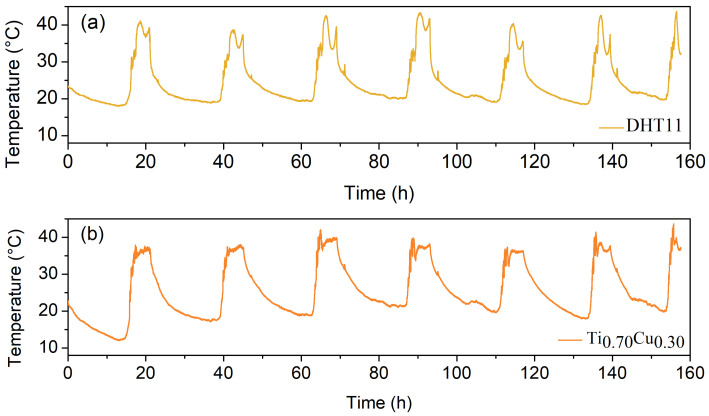
(**a**) Temperature as a function of the time for commercial temperature sensor DHT11 response. (**b**) Temperature measurement with the developed Ti${}_{0.70}$Cu${}_{0.30}$ thin film.

Taking into account the previous results, the Ti${}_{0.70}$Cu${}_{0.30}$ thin film was connected to the electrical circuit presented in Figure 1 to acquire the room temperature signal for 160 h. The results are found in Figure 7a (Ti${}_{0.70}$Cu${}_{0.30}$) and Figure 7b (DHT11).

It is evident the similarity between the developed sensor and the commercial one. The maximum value obtained in both systems is related to noonday, where the two systems were directly exposed to the Sun. In this sense, the present work successfully demonstrates the production and measurement of the temperature using Ti${}_{1-x}$Cu${}_{x}$ thin film, which opens the use of these materials to several applications in which temperature detections are requested.

## 4. Conclusions

A systematic study of the properties of Ti${}_{1-x}$Cu${}_{x}$ thin films was carried out to verify their use as a multifunctional temperature sensor with antimicrobial properties. Irrespective of Cu concentration the Ti${}_{1-x}$Cu${}_{x}$ thin films showed an interesting temperature dependence response with well-defined linearity and stability. However, among the analyzed compositions, it was possible to identify that Ti${}_{0.70}$Cu${}_{0.30}$ has better characteristics to act as RTD, especially the $\alpha_{TCR}$ of 1990 $\times \;10^{-6}$ ${}^{{}^{\circ}}$C${}^{-1}$, and the stability of the response in the interval between room temperature and 100 ${}^{{}^{\circ}}$C. When matched to the commercial DHT11 temperature sensor, the Ti${}_{1-x}$Cu${}_{x}$ thin film showed similar performance in 160 h tested. Furthermore, it has antimicrobial properties that acted to $log_{10}$ reduction for the bacteria *S. aures* and *E. coli*. Therefore, we can place the Ti${}_{0.70}$Cu${}_{0.30}$ as an up-and-coming candidate to act as a multifunctional sensor component in an integrated system for specific applications. This study opens new perspectives to integrating our thin film sensor in a more complex multifunctional system in which other effects can be explored. For instance, spintronics sensors can be based on our thin films to promote an easy and quick path to measure thermal gradients.

## Figures and Tables

**Table 1:** Amount of Cu and Ti (at.%) present in the samples measured by EDS and the thickness of the thin films measured in the cross-section SEM micrographs.

Ti${}_{\mathrm{curr}.}$ (mA)	Cu${}_{\mathrm{curr}.}$ (mA)	Ti (at.%)	Cu (at.%)	Ti${}_{1-x}$Cu${}_{x}$	Thick. (nm)	Rough. (nm)
200	5	0.74	0.26	Ti${}_{0.74}$Cu${}_{0.26}$	46.6	4.8
200	10	0.70	0.30	Ti${}_{0.70}$Cu${}_{0.30}$	39.4	8.0
200	15	0.54	0.46	Ti${}_{0.54}$Cu${}_{0.46}$	53.9	6.5

## Data Availability

The data that support the findings of this study are available from the corresponding author upon reasonable request.

## Supplementary Materials

The following supporting information can be downloaded at: https://www.mdpi.com/article/10.3390/s22197665/s1, Figure S1: Cross-section S.E.M. micrographs for: (a) Ti${}_{0.74}$Cu${}_{0.26}$ thin film; (b) Ti${}_{0.70}$Cu${}_{0.30}$ thin film; (c) Ti${}_{0.54}$Cu${}_{0.46}$ thin film. Figure S2: (a) Electrical resistance measured under temperature cycles for Ti${}_{0.74}$Cu${}_{0.26}$ sample. (b) Electrical resistance stability for 50, 75, and 100 ${}^{{}^{\circ}}$C for periods of 10 minutes for Ti${}_{0.74}$Cu${}_{0.26}$. (c) Similar cycles measurements for Ti${}_{0.54}$Cu${}_{0.46}$. (d) Similar stability resistance for Ti${}_{0.54}$Cu${}_{0.46}$ sample.
