# Supplementary material for: High Performance of Metallic Thin Films for Resistance Temperature Devices with Antimicrobial Properties
Source: Sensors (Basel). 2022 Oct 9;22(19):7665. doi: 10.3390/s22197665 (PMC9570709; doi:10.3390/s22197665)
Supplement: Supplementary file 1 [file sensors-22-07665-s001.zip › sensors-1934183-Supplementary.pdf]

sensors

Article

Supplementary Material
High performance of Metallic Thin Films for Resistance Temperature Devices with Antimicrobial Properties

Arthur L. R. Souza [1,2], Marcio A. Correa [1,2], Felipe Bohn [2], Hélder Castro [1], Margarida M. Fernandes [1], Filipe Vaz [1,3] and Armando Ferreira [1,3,*]

1 Centro de Física das Universidades do Minho e do Porto (CF-UM-UP), Universidade do Minho, 4710-057 Braga, Portugal
2 Departamento de Física, Universidade Federal do Rio Grande do Norte, Natal 59078-900, RN, Brazil
3 LaPMET—Laboratório de Física para Materiais e Tecnologias Emergentes, Universidade do Minho, 4710-057 Braga, Portugal
* Correspondence: armando.f@fisica.uminho.pt

Abstract: Our purpose with this supplementary material is to show the experimental procedure details, the cross-section SEM micrographs, and the thermoresistive response for the $Ti_{0.54}Cu_{0.46}$ and $Ti_{0.74}Cu_{0.26}$ thin films.

check for updates

Citation: Souza, A.L.R.; Correa, M.A.; Bohn, F.; Castro, H.; Fernandes, M.M.; Vaz, F.; Ferreira, A. Supplementary Material High performance of metallic thin films for resistance temperature devices with antimicrobial properties. *Sensors* **2022**, *22*, 7665. https://doi.org/10.3390/s22197665

Academic Editors: Cecilia Cristea and Bogdan Feier

Received: 8 September 2022
Accepted: 5 October 2022
Published: 9 October 2022

Publisher's Note: MDPI stays neutral with regard to jurisdictional claims in published maps and institutional affiliations.

Copyright: © 2022 by the authors. Licensee MDPI, Basel, Switzerland. This article is an open access article distributed under the terms and conditions of the Creative Commons Attribution (CC BY) license (https://creativecommons.org/licenses/by/4.0/).

1. SEM. micrographs

Figure S1 shows the cross-section SEM. micrographs for Ti1-xCux thin films analyzed in this letter. Through cross-section SEM. micrographs, it was possible to determine the thickness of the thin films, 46.6 nm for $Ti_{0.74}Cu_{0.26}$, 39. 4 nm for $Ti_{0.70}Cu_{0.30}$, and 53.9 nm for $Ti_{0.54}Cu_{0.46}$.

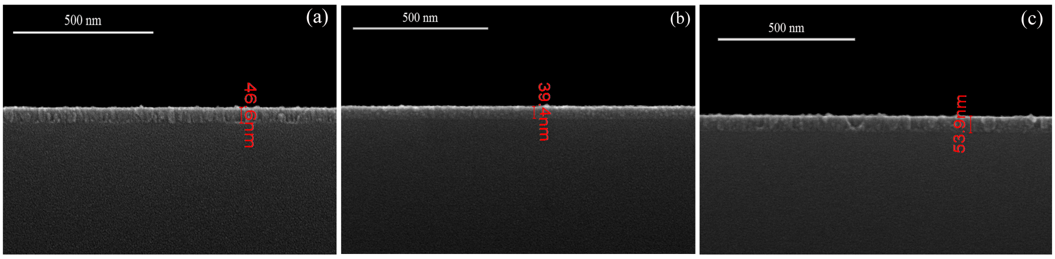

Figure S 1. Cross-section S.E.M. micrographs for: (a) $Ti_{0.74}Cu_{0.26}$ thin film; (b) $Ti_{0.70}Cu_{0.30}$ thin film; (c) $Ti_{0.54}Cu_{0.46}$ thin film.

2. Thermosensitive response

Figure S2 shows the electrical resistance variation to the applied temperature to the $Ti_{0.74}Cu_{0.26}$ and $Ti_{0.54}Cu_{0.46}$ thin films. The $Ti_{0.74}Cu_{0.26}$ film presents the largest variation in the electrical resistance, Figure S2a, among the compositions analyzed in this work, and as a consequence, the highest TCR, see Figure S2c. This is due to the lower copper content present in the film. Although the TCR is high, this composition does not show stability in the thermosensitive response, Figure S2b, compared to $Ti_{0.70}Cu_{0.30}$ film, during the heating and cooling cycles.

The $Ti_{0.54}Cu_{0.46}$ film shows the smallest variation in the electrical resistance, Figure S2c, due to the higher percentage of copper present in the sample, which results in lower TRC in comparison with the $Ti_{0.70}Cu_{0.30}$.

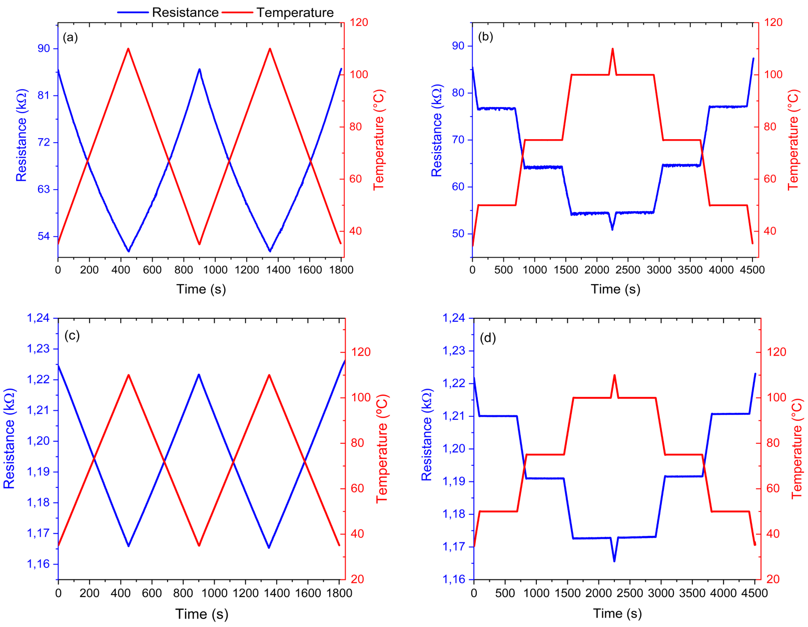

Figure S 2. (a) Electrical resistance measured under temperature cycles for $Ti_{0.74}Cu_{0.26}$ sample. (b) Electrical resistance stability for 50, 75, and 100 °C for periods of 10 minutes for $Ti_{0.74}Cu_{0.26}$. (c) Similar cycles measurements for $Ti_{0.54}Cu_{0.46}$. (d) Similar stability resistance for $Ti_{0.54}Cu_{0.46}$ sample.
